# The Evaluation of Pro-Cognitive and Antiamnestic Properties of Berberine and Magnoflorine Isolated from Barberry Species by Centrifugal Partition Chromatography (CPC), in Relation to QSAR Modelling

**DOI:** 10.3390/ijms18122511

**Published:** 2017-11-24

**Authors:** Wirginia Kukula-Koch, Marta Kruk-Słomka, Katarzyna Stępnik, Radosław Szalak, Grażyna Biała

**Affiliations:** 1 Chair and Department of Pharmacognosy with Medicinal Plant Unit, Medical University in Lublin, 20-093 Lublin, Poland; 2 Department of Pharmacology and Pharmacodynamics, Medical University of Lublin, 20-093 Lublin, Poland; marta.kruk@umlub.pl (M.K.-S.); grazyna.biala@umlub.pl (G.B.); 3 Faculty of Chemistry, Chair of Physical Chemistry, Department of Planar Chromatography, Maria Curie-Skłodowska University, 20-031 Lublin, Poland; katarzyna.stepnik@poczta.umcs.lublin.pl; 4 Department of Animal Anatomy and Histology, Faculty of Veterinary Medicine, University of Life Science, 20-950 Lublin, Poland; radek.szalak@up.lublin.pl

**Keywords:** *Berberis*, alkaloids, passive avoidance test, counter-current chromatography, memory and learning, mice

## Abstract

Civilization diseases associated with memory disorders are important health problems occurring due to a prolonged life span. The manuscript shows the results of an in vivo study targeting the emergence of two drug candidates with anti-amnestic properties. The preceding quantitative structure–activity relationship (QSAR) studies provided information on the ability of berberine and magnoflorine to cross the blood–brain barrier (BBB). In the light of these findings, both compounds were purified from crude plant extracts of barberries: berberine—from *Berberis siberica* using a method published earlier, and magnoflorine—from *Berberis cretica* by centrifugal partition chromatography (solvent system: ethyl acetate:butanol:water-0.6:1.5:3 *v*/*v*/*v*). Both the compounds were evaluated for their memory enhancing and scopolamine inhibitory properties in an in vivo passive avoidance (PA) test on mice towards short-term and long-term memory. Cognition enhancing properties were observed at the following doses: 5 mg/kg (i.p.) for berberine and 20 mg/kg (i.p.) for magnoflorine. In addition, both the tested isoquinolines with the co-administered scopolamine were found to block long-term but not short-term memory impairment. No influence on the locomotor activity was observed for the tested doses. The results confirmed a marked central activity of magnoflorine and showed the necessity to lower the dosage of berberine. Optimized purification conditions have been elaborated for magnoflorine.

## 1. Introduction

Dementia disorders constitute a growing problem affecting inhabitants of developed countries. The advancement of knowledge has contributed to the prolongation of life, which undoubtedly favors various disorders of the central nervous system (CNS) such as Alzheimer’s disease (AD). More than 18 million patients suffer from AD and, according to the World Health Organization (WHO), this number is predicted to grow to as much as ca. 70 million patients by 2050 [1]. An improvement on the efficacy of existing and an introduction of new therapeutic strategies in the treatment of CNS disorders associated with dementia slow the progression of the disease.

Some natural products are being used in the basic treatment of AD [2,3]. Thus, this situation encourages the authors to search for new drug candidates from similar groups of natural products that could possess improved pharmacokinetic and toxicological profiles.

The currently available drugs often bear an increased toxicity or considerable side effects in proportion to the prescribed dosage. In addition, the development of tolerance for their activity has been observed, which triggers the necessity to increase the drug dose and in turn induce the occurrence of side effects.

In a former screening study [1], a marked anticholinesterase activity of two alkaloids: berberine and magnoflorine, in an in vitro thin-layer chromatography (TLC)-based bioautographic study of *Argemone mexicana* extracts was revealed. To confirm these preliminary results, a reference to the in vivo studies on both compounds was needed.

Published in vivo studies of berberine performed after traumatic brain injury in mice showed its neuroprotective properties toward brain as quick as 10 min after administration [4]. This action resulted from the reduction in permeability of leukocytes to the injury site. In addition, rats treated with the extract of *Coptis chinensis*, which contains a high quantity of berberine, showed its antiamnesic action in the scopolamine-induced amnesia model [5]. Another study confirmed its ability to improve spatial memory in rats in a Morris water maze. Hydrochloride salt of berberine at a dose of 50 mg/kg (p.o.) reduced the symptoms of AD, and decreased the synthesis of Interleukin-I β (IL-β) and nitric oxide synthase (iNOS) [6].

In addition, berberine has been documented to increase the viability of cells in the hippocampus and to possess the ability to regenerate neurons located peripherally in the nervous system by an accelerated remyelination of nerve cells, when administered at a dose of 20 mg/kg (i.p.) daily for a week [7].

Scientific reports on the biological activity of magnoflorine are scarce, which is why this compound was of interest to the authors and, in this study, was compared with the activity of a model isoquinoline alkaloid—berberine. Schiff [8] showed a pronounced activity of magnoflorine toward the muscarinic and serotoninergic receptors. This finding encouraged the authors to evaluate its CNS activity further.

To achieve all the goals of the study, in the first step, it was important to check the ability of berberine and magnoflorine to cross the blood–brain barrier (BBB). For this purpose, detailed quantitative structure–activity relationship (QSAR) studies were performed to construct new models of the pharmacological behavior assessment, such as the pharmacokinetic descriptors of the brain, namely, logBB, logPS, logPS*_fu,brain_ fractions unbound in plasma and brain as well as some physicochemical and linear solvation energy relationship (LSER) parameters to further discuss the application of previously obtained in vitro results and about the ability of studied compounds to exert any central effects.

Then, a semi-preparative purification of these molecules by hydrostatic counter-current chromatography was planned and optimized to sustain the need for a high quantity of natural products, which would cover the scheduled dosage in the in vivo study. This technique, however, is characterized by high capacity, low solvent consumption and a possible scale-up, resulting in a one-step purification of high loads of crude samples.

The extracts from *Berberis cretica* and *Berberis siberica*, two deciduous shrubs occuring in South East Europe/Asia Minor and Central Asia, respectively, known to produce isoquinoline alkaloids (including berberine and magnoflorine), were selected for the purification of these two alkaloids due to a higher content of the desired molecules in relation to the previously studied *Argemone mexicana* [1,9].

For the assessment of antiamnestic and pro-cognitive properties of alkaloids, a passive avoidance (PA) test was performed on several groups of mice. In addition, the influence of alkaloids on the locomotor activity of mice for all studied doses was evaluated, in order to obtain the final results on both pharmacological activity and on the ranges of therapeutic windows of both compounds, which are expressed as a disturbance into the mice motility.

## 2. Results

### 2.1. Quantitative Structure–Activity Relationship (QSAR) Studies

To reduce the differences between the actual and the predicted values, the QSAR models were based on the multiple linear regression (MLR) methodology with backward elimination of variables. The best relationship between the BBB descriptors (logBB, logPS) and various physicochemical parameters have been established as a result of many attempts. In each case, the following statistical parameters were determined: determination coefficient, significance level, Fisher criterion, and mean squared error. Then, during the analysis of the above parameters, the best models have been selected (high correlation coefficient, low mean squared error, low *p*-value, etc.).

Based on the parameters from Table 1, some entirely new QSAR models for protoberberine and aporphine alkaloids have been constructed.

Optimization of the structures of the studied alkaloids was conducted before the calculation of both LSER and physicochemical parameters. To provide a structural similarity between the tested compounds, biological activity of the substances should be determined against the chemical group of which they are a part. Therefore, the following compounds have been considered among protoberberine alkaloids: berberine, protoberberine, sanguinarine, coptisine, stylopine, chelidonine, and jatrorrhizine. All of these alkaloids have a 5,6-dihydrodibenzo[*a*,*g*]quinolizinium (C17H14N^+^) skeleton as the basic skeleton of the quaternary protoberberine alkaloids. The group of aporphine alkaloids included: magnoflorine, tetrandrine, glaucine, thalrugosine, protopine, boldine, corydine, and norcorydine. All the above-mentioned compounds follow the Lipiński rule of five [10]; thus, BBB parameters, LSER descriptors, and some physicochemical parameters have been calculated using the ACD/Percepta software (version 2012, Advanced Chemistry Development, Inc., Toronto, ON, Canada).

For protoberberine alkaloids, the following models were obtained:

(1) $$\begin{array}{l} \mathrm{logBB}=-0.433+0.502A-0.543B+0.732S-0.011E-0.392V\\ n=7,R^{2}=95.95\%,S=0.113 \end{array}$$

Considering the chosen structural parameters, that is, lipophic descriptor (logP_o/w_), molar mass (M) and topological polar surface area (TPSA), the rate of penetration into the brain (logPS) has been estimated as follows:

(2) $$\begin{array}{l} \mathrm{logPS}=-1.981+0.6184\;{\mathrm{logP}}_{o/w}-0.003552M+0.00221\mathrm{TPSA}\\ n=7,R^{2}=99.90\%,S=0.414 \end{array}$$

Likewise, the following models have been constructed for aporphine alkaloids:

(3) $$\begin{array}{l} \mathrm{logBB}=-2.34-1.94A-3.98B+1.84S+3.65E-1.41V\\ n=8,R^{2}=74.98\%,S=0.252 \end{array}$$

(4) $$\begin{array}{l} \log\;\mathrm{PS}=-2.118+0.5703\;{\mathrm{logP}}_{o/w}-0.00128M-0.0164\;\mathrm{TPSA}\;\\ n=8,\;R^{2}=96.85\%,\;S=0.244 \end{array}$$

**Figure 1:**
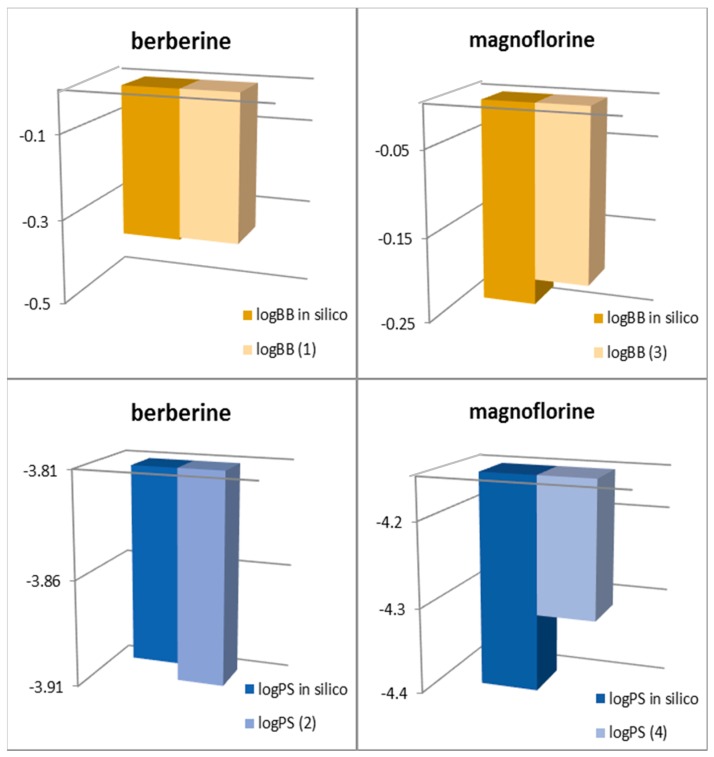
Comparison between the logBB (logPS) calculated in silico and the values obtained from the newly constructed models.

The logPS and logBB values calculated from the newly constructed Models (1)–(4) for berberine and magnoflorine have been compared with the predicted proper values predicted in silico (Figure 1). The values obtained from the newly formed models have been found to be very close to those predicted in silico. This can indicate that the newly formed models are relevant for description of BBB permeability of the studied compounds. The best fit was obtained for Model (1) based on the Abraham parameters.

#### Applicability Domain

The applicability domain has to be essentially defined to evaluate the reliability of the QSAR models. For this purpose, the Williams plot was plotted, illustrating the relationships between standardized residuals and leverages. In our study, standardized residuals are the differences between logBB (logPS) values in silico calculated and predicted from the newly constructed models. All the compounds that fall into that category may be treated as reliable. For each tested compound, the leverage *h_i_* value has been calculated:

(5) $$h_{i}={\left(X^{T}X\right)}^{-1}x_{i}{}^{T}(i\;=\;1,\;...,\;n)$$

where *x_i_* is the row vector of the descriptors, *T* is the matrix/vector transposed, *X* and *i* are the variable matrices deduced from the training set variable values.

Moreover, the critical leverage values h* were estimated for each group of the tested compounds using the following general equation:

(6) $$h^{*}=3\left(k+1\right)/N$$

where *k* is the number of model parameters and *N* is the number of training compounds.

The h* values were calculated for logBB models (for protoberberines = 2.57; aporphines = 2.25). Analogously, h* values for logPS models are as follows: 1.71 (protoberberines) and 1.5 (aporphines). Because all the obtained leverage values for each tested compound (*h_i_* values are less than the critical h* values, and the predicted models can be treated as reliable).

Based on the obtained relations, it was deduced that both berberine and magnoflorine are in the applicability domain of the models.

To check which of the tested structural parameters is the most significant for permeation into the brain, analysis of variance (ANOVA) was performed (Table 2) using the Minitab 17 Statistical Software (Minitab Inc., State College, PA, USA). In the group of aporphine alkaloids, the obtained *p*-values for molar mass and TPSA parameters were found to be greater than the significance level; therefore, the influence of these descriptors on permeation into the brain is statistically insignificant. However, *p*-value for the logP_o/w_ parameters is less than the significance level; therefore, lipophilicity of aporphine alkaloids can play a key role in BBB permeability. Similarly, for protoberberine alkaloids, all of the tested parameters are statistically significant for BBB permeability. The above correlations confirm (according to the Hansch model) that lipophilicity as well as steric and electronic properties of the molecule affect BBB permeability of protoberberine alkaloids.

**Figure 2:**
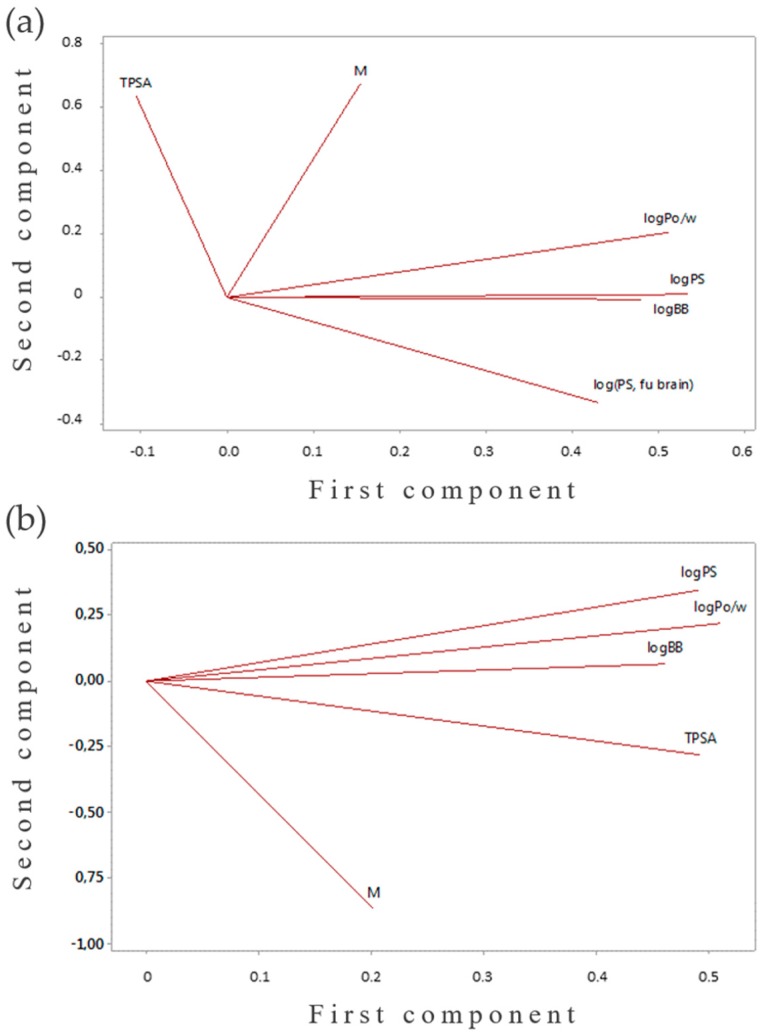
Loading plots of the chosen parameters for: (**a**) aporphine; (**b**) protoberberine alkaloids.

To confirm these assumptions, principal components analysis (PCA) was conducted (Minitab 17 software). The results of PCA were obtained using in silico calculated logBB, logPS, logPS*f_u,brain_, logP_o/w_, molar mass, and TPSA values. The first two principal components explain more than 87% of the variance in the data in both the aporphines and protoberberines groups. In Figure 2, loading plots of the above-mentioned parameters are presented. As we can see from the loading plot corresponding to the first two principal components, there is a correlation between the BBB parameters and logP_o/w_ values for aporphines and between the BBB parameters and all the tested physicochemical descriptors for protoberberines. Therefore, the previous correlations between the BBB descriptors and the chosen molecular parameters were proven. Thereby, the initial in silico BBB studies as well as the newly constructed model and the PCA analysis indicated the ability of both berberine and magnoflorine to cross the BBB.

### 2.2. HPLC and HPLC-MS Analysis of the Extract Composition

A high-resolution mass spectrometer coupled with a liquid chromatograph was used in order to recognize the major secondary metabolites present in a variety of extracts obtained from the selected representatives of Papaveraceae and Berberidaceae in the search for magnoflorine and berberine. Two species were selected as potential sources of these compounds for their isolation for in vivo studies: Cretan barberry (*Berberis cretica*), whose roots contained a high quantity of magnoflorine, and Siberian barberry (*Berberis siberica*) for the purification of berberine in its overground parts, as a successful centrifugal partition chromatography (CPC) separation protocol have been elaborated earlier for berberine by the authors [11]. The overground parts of Siberian barberry were characterized by a high variety of metabolites and a marked content of phenols (a variety of simple phenolic acids and flavonoids such as luteolin and apigenin) present next to alkaloids (e.g., berberine, palmatine, pronuciferine, pakistanamine, and magnoflorine), whereas Cretan barberry contained high quantities of isoquinoline alkaloids, such as an aporphine alkaloid-magnoflorine (which was found to be the major alkaloid in the extract), and protoberberines–berberine, jatrorrhizine, and palmatine, with a low contribution of phenolics (Figure S1) [11].

### 2.3. Purification of Magnoflorine and Berberine from Plant Extracts by CPC Chromatography

According to the rules of counter-current chromatography, the partition coefficient values (expressed as the peak area of a given peak in the upper phase chromatogram, divided by the peak area of the same peak in the lower phase chromatogram) should range around 0.5–2 to provide a successful separation [12]. The authors’ idea was to construct a biphasic solvent system with satisfactory enough *K*-values to achieve a high-purity magnoflorine after a single injection of a total extract into the column. Because of a high polarity of the alkaloid, determined in the initial test tubes’ trials with Arizona system solvents, a water-containing biphasic system had to be considered. Considering the above requirements, the following solvent system was selected for the purification of magnoflorine from Cretan barberry’s root: ethyl acetate:butanol:water (0.6:1.5:3 *v*/*v*/*v*). It delivers the best separation conditions among all tested systems and provides a short settling time, which did not disturb the countercurrent separation at high rotation speeds.

The calculated *K*-values for the major components of the extract in the most favourable solvent system were the following: 0.6 (for magnoflorine), 8.9, 3.4, 2.8, 2.3, and 1.8.

Due to a higher affinity of magnoflorine to the aqueous (lower) phase, the purification was performed in the descending mode of operation, sustaining relatively high rotation speeds and a medium-speed flow rate. The remaining compounds (among them berberine, palmatine, and jatrorhizine) were eluted from the column together with a stationary phase. After the separation, the composition of every second fraction was analyzed on an HPLC chromatograph to join the samples with a similar content. In case of doubts, further injections were performed.

**Figure 3:**
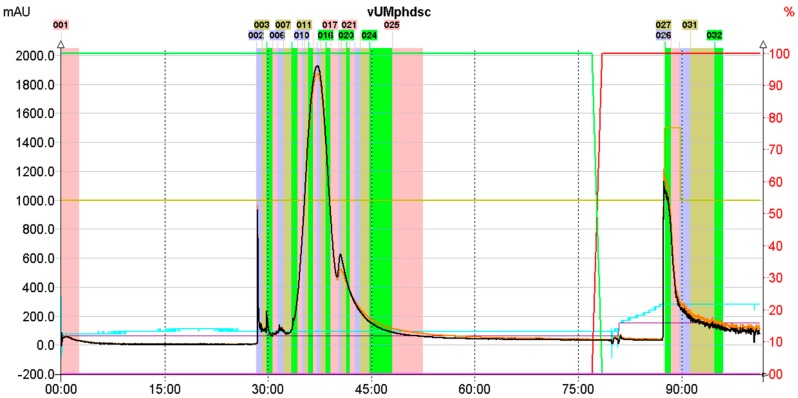
CPC chromatogram obtained from the separation of methanolic extract from Cretan barberry with a prominent magnoflorine peak in the 35th minute (above).

As a consequence, the optimized purification protocol resulted in the collection of magnoflorine from the eluate quickly—in the 35th minute, a purity of 91% directly from the crude extract was obtained (see Figure 3).

To increase the purity of magnoflorine, the resultant fractions containing the alkaloid were mixed, evaporated on a rotary evaporator at 45 °C and transferred into a column filled halfway with Sephadex LH-20, conditioned in a mixture of methanol:water (30:70 *v*/*v*). The residue containing magnoflorine was then washed with the same mixture of solvents. The collected fractions delivered enriched magnoflorine-containing fractions with a purity of 97.4% (see Figure S2).

### 2.4. Locomotor-Activity

#### 2.4.1. The Influence of Berberine on the Locomotor Activity of Mice

**Figure 4:**
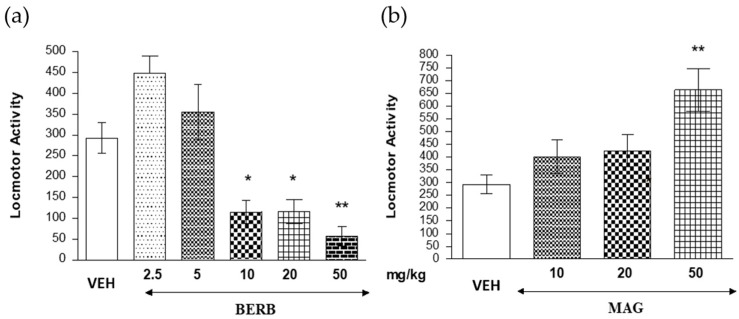
Effects of berberine (BERB) (**a**) and magnoflorine (MAG) (**b**) on the locomotor activity. The data are shown as the means ± standard error of the mean (SEM), photocell beam breaks of mice measured immediately after injection for 60 min; *n* = 9; * *p* < 0.05; ** *p* < 0.01 vs. vehicle (VEH)-treated control group; Tukey’s test.

One-way ANOVA analyses revealed that an acute i.p. administration of berberine (2.5–50 mg/kg) had a statistically significant effect on locomotion of mice (*F*(5.47) = 15.70; *p* < 0.0001). Indeed, the Tukey’s test confirmed that injection of berberine significantly decreased locomotor activity of mice (*p* < 0.05 for the doses of 10 and 20 mg/kg; *p* < 0.01 for the dose of 50 mg/kg) as compared with the control vehicle-injected group (Figure 4).

#### 2.4.2. The Influence of Magnoflorine on the Locomotor Activity of Mice

One-way ANOVA revealed that an acute i.p. administration of magnoflorine (5–20 mg/kg) had a statistically significant effect on locomotion of mice (*F*(3.31) = 5.821; *p* < 0.0032). Indeed, Tukey’s test confirmed that injection of magnoflorine at a dose of 50 mg/kg significantly increased the locomotor activity of mice (*p* < 0.01) as compared with that of the control vehicle-injected group (Figure 4).

### 2.5. Memory-Related Responses

#### 2.5.1. The Influence of Berberine on the Short- and Long-Term Memory Acquisition in the Passive Avoidance (PA) Test in Mice

One-way ANOVA revealed that the administration of acute i.p. doses of berberine (2.5, 5, 10 and 20 mg/kg) had a statistically significant effect on latency index (LI) values for short-term memory acquisition (*F*(4.41) = 2.770; *p* = 0.0413) and long-term memory acquisition (*F*(4.33) = 4.472; *p* = 0.0062).

**Figure 5:**
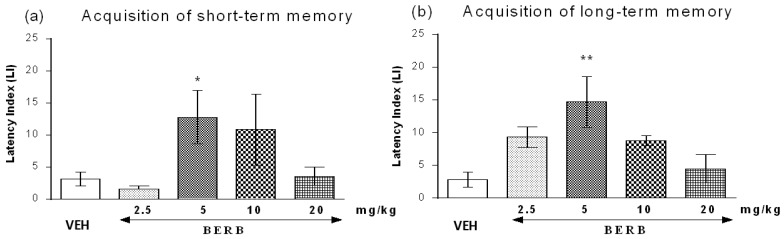
Effects of an acute berberine (BERB) on the latency index (LI) during the short-term or long-term acquisition trials using the PA test in mice. BERB (2.5, 5, 10 and 20 mg/kg; i.p.) or vehicle (VEH) were administered: 30 min before the first trial and animals were re-tested 2 h (for short-term memory (**a**)) or 24 h (for long-term memory (**b**)) later; *n* = 10–11; the means ± SEM; * *p* < 0.05; ** *p* < 0.01 vs. VEH-treated control group; Tukey’s test.

Indeed, treatment with berberine at the dose of 5 mg/kg significantly increased LI values in the PA test in mice compared with those in the vehicle-treated control group (*p* < 0.05, a post-hoc Tukey’s test—for short-term memory acquisition (Figure 5a) and *p* < 0.01, a post-hoc Tukey’s test, for long-term memory acquisition (Figure 5b), indicating that berberine, at the used dosage, improves the short-term as well as long-term acquisition of memory and learning.

#### 2.5.2. The Influence of Magnoflorine on the Short- and Long-Term Memory Acquisition in the PA Test in Mice

The statistical analysis (one-way ANOVA test) showed that an acute i.p. administration of magnoflorine (5, 10, and 20 mg/kg) exhibited a statistically significant effect on the measured LI values calculated for the acquisition of both short-term (*F*(3.30) = 3.491; *p* = 0.0292) and long-term memory (*F*(3.30) = 3.083; *p* = 0.0441).

**Figure 6:**
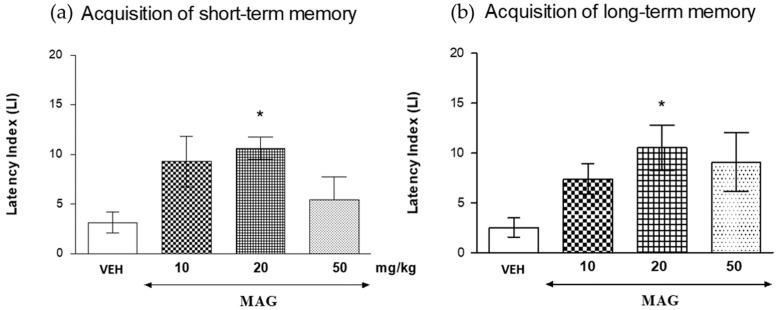
Effects of an acute magnoflorine (MAG) injection on the latency index (LI) during the short-term or long-term acquisition trial using the PA test in mice. MAG (10, 20 and 50 mg/kg; i.p.) or vehicle (VEH) were administered: 30 min before the first trial and animals were re-tested 2 h (for short-term memory (**a**)) or 24 h (for long-term memory (**b**)) later; *n* = 11–12; the means ± SEM; * *p* < 0.05 vs. VEH-treated control group; Tukey’s test.

Indeed, treatment with magnoflorine at the dose of 20 mg/kg significantly increased LI values in the PA test in mice compared with those in the vehicle-treated control group (*p* < 0.05, a post-hoc Tukey’s test—for short-term memory acquisition (Figure 6a) and for long-term memory acquisition (Figure 6b), indicating that magnoflorine at this used dose, improves the short-term as well as long-term acquisition of memory and learning.

Next, based on the results obtained from the above pilot experiments, the non-effective (in PA test) dose of berberine (2.5 mg/kg), magnoflorine (10 mg/kg), as well as the effective (in PA test) dose of berberine (5 mg/kg) and magnoflorine (20 mg/kg) were then chosen for the next behavioral experiment, evaluating the influence of these plant compounds on the memory impairment, provoked by an acute injection of scopolamine (1 mg/kg), using the PA test in mice. The used doses of berberine or magnoflorine had no influence on the locomotion of the mice.

#### 2.5.3. Influence of an Acute Administration of Noneffective and Effective Dose of Berberine on the Memory Impairment Induced by an Acute Administration of Scopolamine in the PA Test in Mice

**Figure 7:**
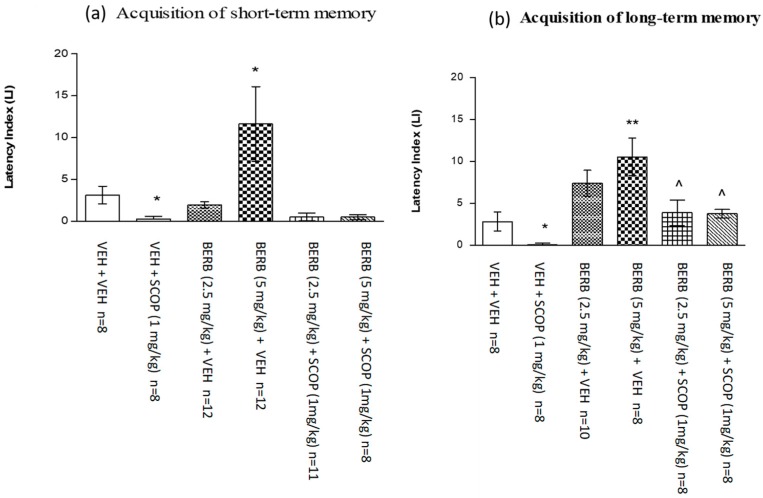
The influence of an acute administration of berberine (BERB) on the memory impairment induced by an acute injection of scopolamine (SCOP), expressed as latency index (LI) during the short-term (**a**) or long-term (**b**) memory acquisition using the PA test in mice. A non-effective dose of BERB (2.5 mg/kg), effective dose of BERB (5 mg/kg) or vehicle were administered 15 min before vehicle or SCOP (1 mg/kg) injection. All drugs were administered 15 min before the first trial and animals were retested 2 h (for short-term memory) or 24 h (for long-term memory) later; *n* = 8–12; the means ± SEM; * *p* < 0.05; ** *p* < 0.01 vs. VEH/VEH-treated group; ^ *p* < 0.05 vs. VEH/SCOP (1 mg/kg)-treated group; Tukey’s test.

For short-term memory acquisition, one-way ANOVA analyses revealed that there is a statistically significant effect on LI values (*F*(5.49) = 5.635; *p* = 0.0004). Post-hoc Tukey’s test confirmed that berberine at a dose of 5 mg/kg significantly increased LI values in mice in the PA test in comparison with the vehicle/vehicle-treated mice, indicating that berberine at this dose improves the acquisition of long-term memory (*p* < 0.05). In addition, scopolamine at the dose of 1 mg/kg significantly decreased LI values in the PA test in comparison with the vehicle/vehicle treated mice, confirming an amnestic effect of this drug (*p* < 0.05) (Figure 7a). Berberine at any dose used did not influence scopolamine-induced disturbances in the acquisition of short-term memory in mice.

For long-term memory acquisition, one-way ANOVA analyses revealed that there is a statistically significant effect on LI values (*F*(5.45) = 7.237; *p* < 0.0001). The post-hoc Tukey’s test confirmed that berberine at the dose of 5 mg/kg significantly increased LI values in mice in the PA test in comparison to the vehicle/vehicle mice, indicating that berberine at this dose used improves acquisition of short-term memory (*p* < 0.01). Additionally, scopolamine at the dose of 1 mg/kg significantly decreased LI values in the PA test in comparison with the vehicle/vehicle treated mice, confirming an amnestic effect of this drug (*p* < 0.05). Furthermore, this amnestic effect provoked by an acute injection of scopolamine (1 mg/kg) was attenuated by non-effective (2.5 mg/kg) (*p* < 0.05) as well as by effective (5 mg/kg) (*p* < 0.05) dose of berberine in comparison with the vehicle/scopolamine (1 mg/kg)-treated mice (Figure 7b).

#### 2.5.4. Influence of the Non-Effective and Effective dose of Magnoflorine on the Memory Impairment Induced by an Acute Administration of Scopolamine in the PA Test in Mice

**Figure 8:**
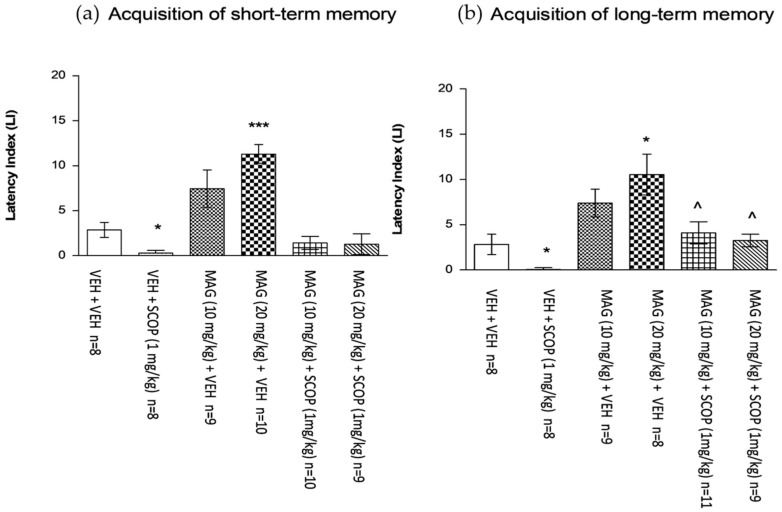
Influence of an acute administration of magnoflorine (MAG) on the memory impairment induced by an acute injection of scopolamine (SCOP), expressed as latency index (LI) during the short-term (**a**) or long-term (**b**) memory acquisition using the PA test in mice. A non-effective dose of MAG (10 mg/kg), effective dose of MAG (20 mg/kg) or vehicle were administered 15 min before vehicle or SCOP (1 mg/kg) injection. All drugs were administered 15 min before the first trial and animals were re-tested 2 h (for short-term memory) or 24 h (for long-term memory) later; *n* = 8–11; the means ± SEM; * *p* < 0.05; *** *p* < 0.001 vs. VEH/VEH-treated group; ^ *p* < 0.05 vs. VEH/SCOP (1 mg/kg)-treated group; Tukey’s test.

For short-term memory acquisition, one-way ANOVA analyses revealed that there is a statistically significant effect on LI values (*F*(5.46) = 14.35; *p* < 0.0001). Post-hoc Tukey’s test confirmed that magnoflorine at a dose of 20 mg/kg significantly increased LI values in mice in the PA test in comparison with the vehicle/vehicle mice, indicating that magnoflorine at this dose improves acquisition of short-term memory (*p* < 0.001). In addition, scopolamine at a dose of 1 mg/kg significantly decreased LI values in the PA test in comparison with the vehicle/vehicle treated mice, confirming an amnestic effect of this drug (*p* < 0.05) (Figure 8a). Similarily to berberine, magnoflorine did not influence scopolamine-induced disturbances in the acquisition of short-term memory in mice at any applied dose.

For long-term memory acquisition, one-way ANOVA analyses revealed that there is a statistically significant effect on LI values (*F*(5.46) = 7.557; *p* < 0.0001). Post-hoc Tukey’s test confirmed that magnoflorine at a dose of 20 mg/kg significantly increased LI values in mice in the PA test in comparison with the vehicle/vehicle mice, indicating that magnoflorine at this dose improves acquisition of long-term memory (*p* < 0.05). In addition, scopolamine at a dose of 1 mg/kg significantly decreased LI values in the PA test in comparison with the vehicle/vehicle treated mice, confirming an amnestic effect of this drug (*p* < 0.05). Furthermore, this amnestic effect provoked by an acute injection of scopolamine (1 mg/kg) was attenuated by a non-effective (10 mg/kg) (*p* < 0.05) as well as by an effective (20 mg/kg) (*p* < 0.05) dose of magnoflorine in comparison with the vehicle/scopolamine (1 mg/kg)-treated mice (Figure 8b).

## 3. Discussion

### 3.1. QSAR Studies

The QSAR used in studying the dependence between the structures and a wide range of compounds’ properties, plays an important role in different fields of sciences including drug design, toxicology, etc. In a QSAR approach, a biological activity results from various compound–receptor interactions accompanying the transport of an active compound through biological membranes [13]. These interactions are assumed to be governed by the chemical structure characterized by lipophilic, steric and electronic parameters of a compound according to the Hansch approach. The QSAR also uses LSER as well as the empirical model of Abraham. This model describes different physicochemical processes in living organisms including blood–brain barrier permeability, which was evaluated for the sake of this study [14,15].

The Hansch approach [16,17] being the property–property relationship model [18,19,20,21,22,23], correlates the biological activity of a compound with its physicochemical properties by linear or nonlinear regression analysis.

The general model proposed by Hansch involves biological response of a substance, which can be expressed through physicochemical properties [18]:

(7) $$\frac{d\left(BR\right)}{dt}=ae^{-\frac{{\left(\log P-\log P_{o}\right)}^{2}}{b}}\left(C\right)\left(k_{1}\right)$$

where:

*BR*—the biological response in a constant time interval;
*C*—the molar concentration of compound producing a standard response in a constant time interval;
*k*_1_—the coefficient determined by the method of least squares;
*a*, *b*—the constants;
logP_o/w_—the logarithm of n-octanol/water partition coefficient of a derivative *d*log(1/*C*)/dlogP = 0.

Biological activities of compounds are characterized by some basic intermolecular interaction forces: mainly steric, hydrophobic, and electronic effects. Therefore, the LSER model tends to describe different biological and physicochemical processes. The general LSER equation originally used by [24,25,26] is expressed as follows:

(8) SP=c+vV+sS+bB+aA+eE

where *SP* is the dependent solute property in a given system. The independent variables are solute descriptors: *V* is the solute McGowan volume in units of cm^3^ mol^−1^/100, *S* is the polarizability/dipolarity, *B* is the overall hydrogen–bond basicity, *A* is the overall hydrogen–bond acidity, and *E* is an excess molar refraction. The coefficients: *v*, *s*, *b*, *a*, *e* reflect the differences in the two phases between which the compound is transferred.

In the presented paper, the QSAR studies were applied to determine the BBB penetration of both alkaloids, which was expressed as the logarithm of the ratio between the brain and blood concentrations of the tested substances [13]:

(9) log BB=log(conc. in brain/conc. in blood)

To investigate BBB permeability more thoroughly, the most important pharmacokinetic parameters of brain, namely, the BBB permeability–surface area product (PS), usually given as the logPS, the brain–plasma equilibration rate (logPS*f_u,brain_) and the fractions unbound in plasma and brain have been calculated (with ACD/Percepta software). The obtained BBB permeability parameters as well as LSER ones were collated with the most important physicochemical descriptors (Table 1).

In addition, other isoquinoline alkaloids tested, which belonged to the group of aporphines and protoberberines, were found to exhibit the ability to penetrate the BBB. These properties may depend on physicochemical characteristics of a molecule i.e., lipophilicity, ionization state, molecular volume, molecular mass, and so on. These characteristics are important determinants of distribution and potential accumulation of a substance within brain tissues. Physicochemical characteristics can impact cellular and subcellular distribution e.g., entry into the CNS via the BBB [27].

The performed calculations clearly showed that both selected alkaloids—berberine and magnoflorine—are prone to penetrate the BBB and exhibit some pharmacological properties. These findings let the authors continue the studies on the purification and testing of the selected alkaloids.

However, it must be stated that the performance of all newly-constructed QSAR models depends on the availability and quality of experimental in vivo data, which are, in fact, rarely available. In vivo datasets are dependent on various factors like solubility and stability of a compound, detection technique of BBB penetration, dose and compound formulation [28]. To apply QSAR methodology, chemicals located within the applicability domain of the model need to have an appropriate number of measured experimental values of certain biological activity (in our case: BBB permeability). Therefore, in our study, the limitation of the models that we proposed should be pointed out. Among them, unavailability of sufficiently large datasets of both logBB and logPS values are worth mentioning. In addition, it is necessary to remember that logBB values reflect also processes other than BBB permeability including plasma protein binding, tissue binding, local metabolism and others [29]. As regards logPS values, being considered the most distinctive in vivo measure of BBB permeability, it should be emphasized that logPS does not provide information on the substance concentration in the brain [28]. Due to the complex nature of the brain penetration process, it is required to treat in silico obtained values as a kind of modeling assumption. Nevertheless, our in vivo findings that both compounds provide anti-amnesic and pro-cognitive properties suggest that they both cross the BBB as our QSAR model predicted.

### 3.2. The Purification of Alkaloids by CPC Chromatography

During the presented research, both magnoflorine and berberine were successfully purified using counter-current chromatography in sufficient quantities for behavioral studies and a purity exceeding 90%.

A proper separation was influenced by a number of parameters. The most important of them included a good selection of solvent system, but also proper column rotation speed and flow rate settings, which provided an equilibrium between the stationary and mobile phase volumes on the column (good retention of the stationary phase) [30]. To a large extent, the latter parameter depends on how soon after mixing the selected solvent system reaches equilibrium again and creates two separate, immiscible layers [31].

For Cretan barberry, the results of this study clearly indicated that magnoflorine was strongly distributed in the mobile aqueous phase, contrary to other alkaloids present in the extract. This is why it could be easily obtained purified, directly from the crude extract. In addition, the applied parameters enabled high sample loads (1 g of an injected extract). Berberine collected from Siberian barberry aerial parts was washed out in a pH-zone refining mode counter-current chomatography, according to a previous publication of the authors [11]. This mode of operation characterized by the addition of a base to organic phase and an acid to water phase uses the ability of alkaloids to exist in two forms—a nonpolar base and a polar salt, which helps in their purification from other metabolites.

### 3.3. In Vivo Studies

As mentioned in the Introduction section, the tested compounds had been previously found to be acetylcholinesterase inhibitors in in vitro tests [1]. The aim of the study was to assess the influence of berberine and magnoflorine on the acquisition of memory in PA test. In addition, their potency against memory disturbances provoked by scopolamine was tested.

First, the initial locomotor activity tests were performed using a variety of berberine and magnoflorine dosages to well determine the highest non-active and the lowest active dosages. For this purpose, 2.5, 5, 10, 20 and 50 mg/kg (i.p.) of berberine and 5, 10, 20, 50 mg/kg (i.p.) of magnoflorine were administered and the animals reactions were measured in the actometer cages. Based on the results of locomotor activity tests, the doses that did not affect the locomotor activity of animals were selected, to be sure, that no disturbance would affect the results of memory tests. The applied PA test was finally conducted on two selected lowest doses: 2.5 and 5 mg/kg (i.p.) of berberine, and 10 and 20 mg/kg (i.p.) of magnoflorine.

Other studies on berberine showed its memory enhancing activity when administered to rats or mice [13]; however, the majority of them were performed using higher doses of berberine, for example, 10, or even 20 mg/kg (i.p.) [7,31]. This matter concerns the authors as this dosage seems to be too high; in this study, the 10 or 20 mg/kg berberine aggravated the locomotor activity of the tested mice [32].

PA test performed for berberine clearly demonstrated that already a single injection of effective (5 mg/kg) and non-effective (2.5 mg/kg) dose showed the memory enhancing effects; however, the response was stronger for the long-term memory than for the short-term memory after one injection.

With the influence of scopolamine in all the tested groups, the animals were found to be excessively excited, which was reflected in an increased locomotor activity that does not influence cognitive performance. This behavior is induced by the ability of scopolamine to block muscarinic receptors and, based on this ability, evoke disturbed strategy elaboration and place recognition in animals. Those results are consistent with some previous experiments of the authors [33].

The dose of 5 mg/kg of berberine was able to overcome the action of the co-administered scopolamine in the scopolamine model of memory impairment. However, higher doses of this alkaloid did not increase the LI values, which may be connected with the disturbances with previously studied locomotor activity.

Berberine, which had been proven to exhibit memory enhancing properties, was treated as a model compound for the initial tests of the other compound—magnoflorine. This aporphine alkaloid was found less toxic and did not interfere with the locomotor activity of mice at a lower non-effective dose of 10 mg/kg or a higher effective dose of 20 mg/kg, which may indicate that the alkaloid is well tolerated at these selected doses.

Magnoflorine at a dose of 20 mg/kg, improved the cognitive processes of short and long term memory in mice in the PA test significantly, as it increased the passage time of animals to the dark room during the applied test. The animals associated the dark room with a negative electric stimulus, which explains their prolonged stay in a bright room and unwillingness to enter the dark one. Only a single dose of this alkaloid (20 mg/kg, i.p.) improved the cognitive function, assessed on the same day—2 h after the training session but also 24 h after the test, which confirmed the effect of magnoflorine on both short- and long-term memory processes and prevented the animals from entering the dark compartment. A dosage of 10 mg/kg exhibited a similar pro-cognitive tendency; however, the obtained results were not statistically significant. The effect of this dose might be observed in the long-term administration studies, which will be performed by the authors in the near future. In addition, this compound was found to reverse the scopolamine-induced long-term memory impairment, both at a dose of 10 and 20 mg/kg, compared with the control group, which demonstrates the anti-amnestic properties of magnoflorine.

Considering the effect of magnoflorine on acetylcholinesterase assessed earlier [1] and the current behavioral studies, it may be suggested that the effect of cholinergic transmission and related cognitive processes is strongly influenced by this compound. Although magnoflorine may act as an inhibitor of scopolamine-induced memory effects, its probable mechanism of action may be based on the inhibition of acetylcholinesterase. Even though these mechanisms of action are probable, the authors cannot exclude other activities of this compound.

This is the first time the procognitive activity of this compound was evaluated. The obtained results confirm the proprioceptive effects of magnoflorine and qualify it for further pharmacological studies, as, possibly in the future, it could be perceived as a drug candidate directed to the therapeutical strategies of various diseases progressing with memory deficits such as dementia, or AD.

An interesting phenomenon was observed for the tested compounds. Their influence on long-term memory was stronger than on the short-term memory. This might have been due to the actual activity of their metabolites, which appear within a longer time. According to Wang and co-investigators [34], berberine forms a variety of metabolites in vivo during its demethylation, demethylenation, reduction, hydroxylation and conjugation in vivo. In fact, the metabolites present in the forms of glucuronides or sulfates are more abundant in blood samples and more active than the original compound. A similar occurrence might be observed for magnoflorine.

## 4. Methods

### 4.1. Chemicals

All gradient grade solvents were obtained from Avantor Performance Materials (Center Valley, PA, USA). Chromatographic and spectroscopic grade solvents used in the quality assessment procedures: acetonitrile, water, acetic acid and formic acid were produced by J.T. Baker (Center Valley, PA, USA). Scopolamine was purchased from Sigma Aldrich (St. Louis, MO, USA).

### 4.2. Plant Material

The root of *Berberis cretica* and the overground parts of *Berberis siberica* were used for the purification of alkaloids for the bioactivity studies. The former was collected and identified by Dr. Eleftherios Kalpoutzakis from the University of Athens in the central part of Crete Island in the fall of 2007. The latter species was obtained and identified by Dr. Ottonbataar Urjin in September of 2015 in the Bayan Province in Mongolia. The gathered plant material was roughly cut, dried at a temperature of 40 °C, later ground in a mortar and directed to extraction. Voucher specimens of both species are stored in the Chair and Department of Pharmacognosy of the Medical University of Lublin, Poland.

### 4.3. Extraction of Plant Material

The extraction of plant material was performed by accelerated solvent extraction in 100 mL stainless steel vessels on 150 g of powdered roots of both species, according to the previously published methods [1,28,35], as described in the Supplementary file.

### 4.4. Identification of Major Constituents of the Extracts by HPLC and HPLC-MS

The composition of both extracts and fractions obtained in the separation process was constantly monitored to provide data on the purification efficiency of the applied techniques. First, the major secondary metabolites—alkaloids and phenolics—present in the extracts were recognized in a high-resolution mass spectrometry analysis, according to the previous studies of the authors [11,35]. Later, based on the obtained HPLC-MS data, the purity control was performed by HPLC for every second fraction from CPC purification. Similar fractions were joined. In addition, HPLC chromatograms delivered suitable information for the calculations of partition coefficient values (*K*) in the optimization of the counter-current separation method. Detailed chromatographic and spectrometric conditions are presented in the Supplementary file. They are based on the previous studies of authors [35].

### 4.5. Purification of Alkaloids by Means of Hydrostatic Counter-Current Chromatography

Centrifugal partition chromatography (CPC) was applied in the separation process of two alkaloids—berberine and magnoflorine. The former was purified from *Berberis siberica* overground parts according to the previously published method [11], whereas the latter was from the powdered root of *Berberis cretica* in an optimized separation protocol described in the scientific literature for the first time.

#### 4.5.1. Purification of Magnoflorine

The CPC separation was performed using an Armen SCPC-250-L instrument (Brittany, France), equipped in a 250 mL rotor, a quaternary pump, a UV detector (DAD 600 Flesh06S), and an automatic fraction collector (LS-5600). Prior to the analysis, a mixture of solvents was freshly prepared in a large separating funnel. The upper and lower phases were collected to separate amber glass bottles. The following solvent system was selected for the fractionation of Cretan barberry’s root extract: ethyl acetate:butanol:water (0.6:1.5:3 *v*/*v*/*v*), which was pumped into the rotating column in the descending mode at the rotation speed of 1300 rpm. The solvent flow was set at 6 mL/min until the end of the process—for 150 min. After 90 min, the stationary phase was pumped in the same operation mode to elute the components with a higher affinity to the stationary phase. In addition, 1 g of extract dissolved in 5 mL of a 50:50 mixture of upper and lower phases was introduced immediately after the column was filled with a stationary phase. Subsequent injections were made one by one, without stopping the column’s rotations.

#### 4.5.2. Separation of Berberine

Berberine was obtained for the study in a pH-refining mode of CPC chromatography according to the previously described protocol [11], using a biphasic solvent system composed of methyl-*tert*-buthyl ether (MtBE) and water (1:1 *v*/*v*) with the addition of modifiers—10 mM of HCl and 10 mM of trimethylamine (TEA) into the aqueous and organic phases, respectively.

The separation was carried out in an ascending mode in the flow rate of 5 mL/min, rotation speed of 1050 rpm within 220 min.

### 4.6. Animals

A group of naïve male Swiss mice were used in the scheduled experiments (Farm of Laboratory Animals, Warszawa, Poland). Each of them weighted ca. 20–30 g. Standard laboratory conditions were applied to maintain the animals (12-h light/dark cycle, controlled room temperature at 21 ± 1 °C). They were provided free access to laboratory chow (Agropol, Motycz, Poland) and tap water. Before the study, they were acquainted with their home cages for a period of one week. Each experimental group contained 8–12 animals. The behavioral experiments were conducted regularly between 8:00 and 15:00, according to the National Institute of Health Guidelines for the Care and Use of Laboratory, and also based on the directions of Animals and to the European Community Council Directive for the Care and Use of laboratory animals of 22 September 2010 (2010/63/EU). The performed tests were first approved by the Ethics Committee at Medical University of Lublin, Poland.

### 4.7. Drugs

The compounds tested were:

Berberine (2.5, 5, 10, 20, 50 mg/kg) from methanolic extract of *Berberis siberica* herb, and magnoflorine (5, 10, 50 mg/kg)—obtained from methanolic extract of *Berberis cretica* root.

Scopolamine hydrochloride (1 mg/kg) was produced by Sigma-Aldrich (St. Louis, MO, USA).

Magnoflorine, berberine and scopolamine used for the study were dissolved in 0.9% NaCl with an addition of 0.2% of DMSO (vehicle). The tested compound was administered i.p. at a constant volume of 10 mL/kg. Each day prior to the experimentation, the drug solutions were prepared. In parallel, a control group of mice was given i.p. injections of saline with an addition of 0.2% DMSO (later referred to as vehicle), at the very same volume as tested drugs.

### 4.8. Experimental Procedures

Experimental doses of drugs used and procedures were selected based on the literature data [6,7] and our preliminary studies. For each stage of memory as well for both short-term and long-term memory, independent and different groups of mice were used.

#### 4.8.1. Locomotor Activity

The locomotor activity test was recorded for each tested animal in a separate actometer cage (Multiserv, Lublin, Poland; 32 cm in diameter, two light beams) in a low-noise room. In the apparatus, two separate photocell beams, which were located perpendicularily to each other, measured the movements of animals [33]. Horizontal locomotor activity was measured immediately after a single injection of tested drugs, berberine (2.5–50 mg/kg, i.p.), or magnoflorine (5–20 mg/kg, i.p.), scopolamine (1 mg/kg, i.p.) or vehicle for the control group, immediately after the last injection for 60 min.

#### 4.8.2. Memory Related Responses

The applied PA test measured the memory-related responses in the apparatus, which contained two compartments—a lightened one (10 × 13 × 15 cm) and a darkened one (25 × 20 × 15 cm). Fluorescent light (8 W) was illuminating the light chamber. The dark compartment contained an electric grid floor. The entrance of an animal triggered an electric foot shock for 2 s (0.2 mA).

First, a pre-test was arranged to acknowledge the animals with the apparatus. Each mouse was individually introduced to the PA apparatus and allowed to explore the bright compartment for 30 s. Later, a guillotine was raised to let the animals escape to the dark room. When in the dark room, the guillotine door was closed and immediately an electric foot shock at the duration of 2 s (0.2 mA) was applied on the grid floor. The time measured until the opening of the guillotine door to the entrance of an animal to the dark compartment was measured and calculated as latency time (TL1). If the time was longer than 300 s, the experiment was stopped and the animal was transferred to the dark compartment and delivered an electric shock. The time of 300 s was put down in this case.

Then, another trial was conducted and called retention. After a 30 s time for adaptation, the guillotine door were opened in the PA apparatus and the time needed for the animal to enter the dark compartment was measured as TL2. No foot-shock was given in this trial. In addition, 300 s were noted for the animals, which did not move from the light compartment to the dark one.

The PA test enabled the measurement of both short-term and long-term memory, based on the time passed from the pre-test to the actual retention test. Short-term memory was assessed, when the trial was performed 2 h after the training test. Latency time measured after 24 h delivered information on the long-memory process assessment. In both cases, the drugs were administered before the pre-test, and they were expected to interfere with the physiological acquisition of information [33].

The first step of experiment was designed to estimate the influence of tested drugs, berberine and magnoflorine on the acquisition of short- as well as long-term memory in mice, using the PA test.

Berberine (2.5–20 mg/kg, i.p.), magnoflorine (5–20 mg/kg, i.p.), or vehicle, for the control group, were administered 30 min before the first trial and re-tested after 2 h (short-term memory) or after 24 h (long-term memory).

Finally, we evaluated the impact of the combining administration of tested drugs, berberine and magnoflorine on the memory impairment induced by an acute administration of scopolamine.

Berberine (2.5–20 mg/kg, i.p.), magnoflorine (5–20, i.p.) or vehicle, for the control group, were administered 15 min before injection of scopolamine (1 mg/kg, i.p.) or vehicle. Fifteen minutes after the last injection, the mice were tested in the PA test (first trial) and re-tested 2 h (short-term memory) or 24 h (long-term memory) later.

### 4.9. Statistical Analysis

The statistical analysis was performed using one-way analysis of variance (ANOVA). Post-hoc comparison of means was carried out with the Tukey’s test for multiple comparisons when appropriate. The data were considered statistically significant at a confidence limit of *p* < 0.05. ANOVA analysis with a Tukey’s post-hoc test were performed using GraphPad Prism version 5.00 for Windows (GraphPad Software, San Diego, CA, USA). Moreover, to check the usefulness of newly constructed QSAR models, the Principal Components Analysis (PCA) has been done (Minitab 17).

For the locomotor activity, the number of photocell beam breaks was measured for 60 min and put down [33].

For the memory-related behaviors, the results obtained in the PA test were expressed as latency index (LI). It is expressed as the difference between the time measured in the second test (retention test) and the previous test (pre-test, training test) for each animal: LI = TL2 − TL1/TL1.

TL1—the time needed to enter the dark compartment in the training session (pre-test); TL2—the time needed to re-enter the dark part of the apparatus during the retention test [36].

## 5. Conclusions

Berberine and magnoflorine were successfully isolated from two selected barberry species by centrifugal partition chromatography at high purity. They were found to exhibit antiamnestic and cognition enhancing properties in the PA test on mice at the doses of 5 and 20 mg/kg, respectively, towards long-term and short-term memory. No impact on the locomotor activity of mice in the studied dosage was noted, which shows that the selected doses were within the therapeutic windows of both alkaloids. The possible mechanism of action of these two compounds may be related to the inhibition of acetylcholinesterase, as it has been proven in the earlier in vitro studies of the authors. In addition, both compounds block long-term but not short-term memory impairment affected by scopolamine.

## Figures and Tables

**Table 1:** The collation of the blood–brain barrier (BBB) pharmacokinetic parameters, the Abraham linear solvation energy relationship (LSER) descriptors and the chosen physicochemical data.

Compound	logBB	logPS	logPS*_fu,brain_	Fraction Unbound in Plasma	Fraction Unbound in Brain	Molar Mass (M)	Topological Polar Surface Area (TPSA)	Logarithm of n-Octanol-Water Partition Coefficient (logP_o/w_)	Abraham LSER Descriptors				
									A	B	S	E	V
Berberine	−0.35	−3.9	−3.95	0.44	0.98	336.366	40.80	−1.33	0	1.09	2.25	2.25	3957
Magnoflorine	−0.23	−4.4	−4.4	0.58	0.98	342.414	58.92	−1.38	0.55	0.94	1.62	1.62	2.5903

**Table 2:** Statistical *p*- and *F*-values obtained for the tested alkaloids.

Parameter	Aporphines		Protoberberines	
	*F*-Value	*p*-Value	*F*-Value	*p*-Value
logP_o/w_	94.87	0.001	1010.13	0.000
M	1.50	0.288	1405.09	0.000
TPSA	1.86	0.245	46.28	0.0006

## Supplementary Materials

Supplementary materials can be found at www.mdpi.com/1422-0067/18/12/2511/s1.

## Abbreviations

AChE	Acetylcholinesterase
AD	Alzheimer’s Disease
ASE	Accelerated Solvent Extraction
BBB	Blood–Brain Barrier
CC	Column Chromatography
CNS	Central Nervous System
CPC	Centrifugal Partition Chromatography
ESI	Electrospray Ionization
HPLC	High Performance Liquid Chromatography
i.p.	Intraperitoneally
iNOS	Nitric Oxide Synthase
K	Partition Coefficient
LI	Latency Index
LSER	Linear Solvation Energy Relationship
MS	Mass Spectrometry
PA	Passive Avoidance Test
QSAR	Quantitative Structure–Activity Relationship
TEA	Trimethylamine
TL	Latency Time
TPSA	Topological Polar Surface Area
