# Supplementary material for: The Evaluation of Pro-Cognitive and Antiamnestic Properties of Berberine and Magnoflorine Isolated from Barberry Species by Centrifugal Partition Chromatography (CPC), in Relation to QSAR Modelling
Source: Int J Mol Sci. 2017 Nov 24;18(12):2511. doi: 10.3390/ijms18122511 (PMC5751114; doi:10.3390/ijms18122511)
Supplement: Supplementary file 1 [file ijms-18-02511-s001.pdf]

International Journal of
Molecular Sciences

MDPI

The Evaluation of Pro-cognitive and Antiamnestic Properties of Berberine and Magnoflorine Isolated From Barberry Species by Centrifugal Partition Chromatography (CPC), in Relation to QSAR Modelling

Wirginia Kukuła-Koch, Marta Kruk-Słomka, Katarzyna Stępnik, Radosław Szalak and Grażyna Biała

Methods

Extraction of plant material

Accelerated solvent extraction by ASE 100 apparatus (Dionex, USA) was performed in 100 mL stainless steel vessels on 150 g of powdered roots of both species, divided into ca. 30 g portions. Extraction cell was heated in an oven each time and an extractant was pumped at the temperature of 100 ^{0}C according to the previously published method [2].

For *Berberis cretica*, methanol extract was obtained from the powder initially purified with SFE extractor according to the previously published method [3], subjected to ASE extraction at 100 ^{0}C.

The obtained extracts were collected and evaporated to dryness on a rotary evaporator at a temperature of 45 ^{0}C and the oily residue was stored in the amber bottles in a refrigerator until the separation.

Identification of major constituents of the extracts by HPLC and HPLC-MS

Agilent Technologies (Santa Clara, USA) mass spectrometer (G6530B) composed of a quadrupole and a time-of-flight detector with an ESI ionization source, coupled with an LC system (1260 Series) equipped in a pump (G1312C), an autosampler (G132B), a degasser (G1322A), an UV PDA detector (G1315D) and an oven (G1316A) delivered the spectra in a tailored method on an RP-18 chromatographic column (Zorbax Stable Bond, Agilent Technologies, 150 x 2.1 mm, d = 3.5 μm) in the gradient of solvents published previously by the authors [1]. The following mass spectrometer settings were used: capillary voltage 3.5 kV, fragmentor voltage 150 V, skimmer voltage: 65 V, collision energies: 20 and 30 V, gas and sheath gas temperatures: 350 ^{o}C, drying gas and sheath gas flows: 12 L/min each, nebulizer pressure: 35 psig. HPLC chromatograms were recorded using a Shimadzu apparatus (Japan) with a quaternary pump (LC-20AD), a PDA detector (SPD-M20A), an autosampler (SIL-20A) and a degasser (DGU-20A), using a Supelco (Sigma Aldrich) RP-18 column (250 x 4.0 mm, internal diameter: 5 μm) in a gradient of acetonitrile in water in addition of 2% acetic acid previously optimized for isoquinoline alkaloids and published by Kukula-Koch and co-investigators [3].

The evaluation of partition coefficient values

First, a test tube test was performed on methanolic extract from the roots of Cretan barberry to select a proper biphasic system for the purification of magnoflorine – an aporphine alkaloid which is present in this particular extract in a high quantity. For this purpose, the Arizona system table was used to evaluate the polarity of the extract's constituents. As magnoflorine was found to be polar, based on the available scientific literature and considering the Sorensen's diagrams, further biphasic solvent systems were elaborated. The evaluation was performed as follows: a biphasic system in the volume of 5 mL was prepared in a test tube and was left for the separation of upper and lower phases (not longer than 30 s). Ca. 5 mg of a dried extract was introduced to the test tube on a glass spoon and thoroughly mixed for 1 min to provide good dissolution of the sample and distribution between

upper and lower phases. The systems were left for 30 s to obtain two phases. Those which created an emulsion were rejected at this stage. Later the upper and the lower phases were separately filtered through a nylon syringe filter (Cronus, pore diameter 0.45 μm) and were transferred to glass vials. The content of the vials was dried in a vacuum drier (Eppendorff condenser) at a temperature of 40 °C and re-dissolved in 1 mL of methanol (chromatographic grade) prior to further HPLC evaluation.

The determination of the partition coefficient values (*K*) for each major component of the extract was performed through the quantitative analysis of upper and lower phases' content - separately on an HPLC chromatograph. The *K* values for each peak in the obtained chromatograms were expressed as their peak area in the lower phase, divided by their content in the upper phase. Table S1 presents the evaluated biphasic solvent systems for magnoflorine. The results determined the efficiency of the constructed biphasic systems in the purification of secondary metabolites from the extract.

Table S1 The composition of solvent systems evaluated in the separation of Cretan barberry extract

No	Solvent system	Solvents ratio	Remarks
1	n-Hexane-butanol-ethanol-water	4:11:6:15	Similar colour in both phases; the sample verified by HPLC
2	n-Hexane-butanol-ethanol-water	6:9:6:10	Similar colour in both phases; the sample verified by HPLC
3	n-Hexane-butanol-ethanol-water	1:14:6:15	Similar colour in both phases; the sample verified by HPLC
4	MtBE- butanol-ethanol-water	9:5:5:10	The whole extract in the lower phase
5	Butanol-acetic acid-water	15:1:15	Similar colour in both phases; the sample verified by HPLC
6	Butanol-water	1:1	Both phases coloured, higher quantity in the lower phase
7	Butanol-1%hydrochloric acid	1:1	Similar colour in both phases; the sample verified by HPLC
8	Ethyl acetate-butanol-water	0,4:1,6:2	Both phases coloured; higher quantity of the extract in the upper phase; HPLC evaluation
9	MtBE-acetonitrile-water	2:2:3	Similar colour in both phases; the sample verified by HPLC
10	MtBE-acetonitryl-1%hydrochloric acid	2:2:3	Similar colour in both phases; the sample verified by HPLC
11	Hexane-ethyl acetate-methanol-water	1:5:1:5	Lower phase coloured
12	n-Butanol- MtBE-water	10:4:2	Both phases coloured; higher concentration in the lower phase
13	MtBE- hexane-methanol-water	5:1:4:5	No further evaluation

Figure S1. The TIC chromatogram obtained for the root extract from Cretan barberry in the positive ionization mode with the recognized major components.

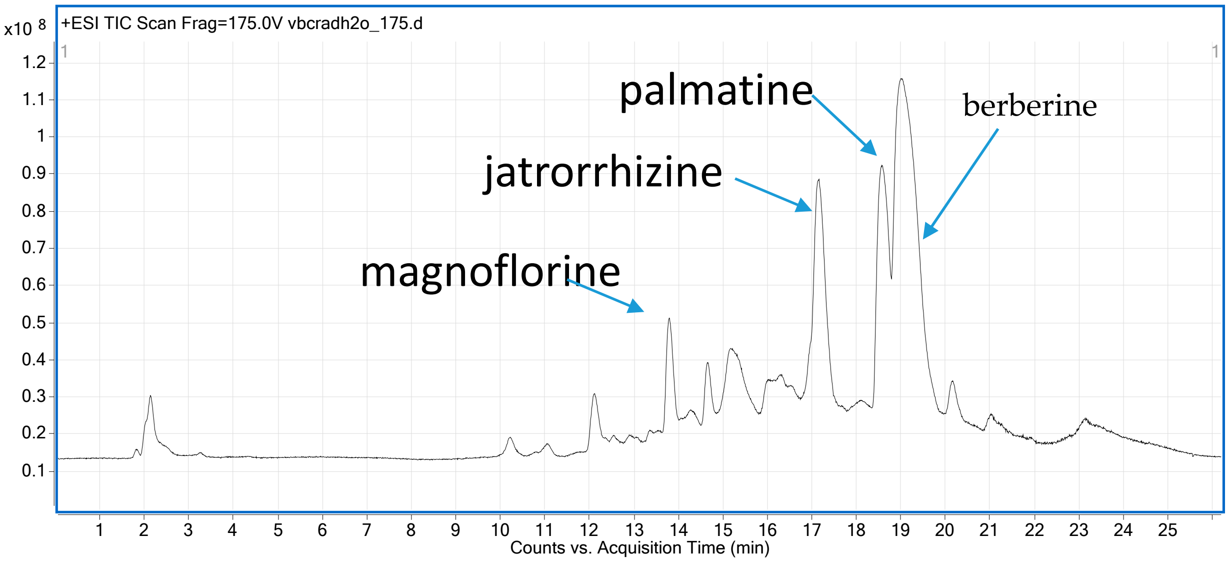

Figure S2. An HPLC chromatogram of purified magnoflorine (recorded in 280nm) collected from CPC and a Sephadex column.

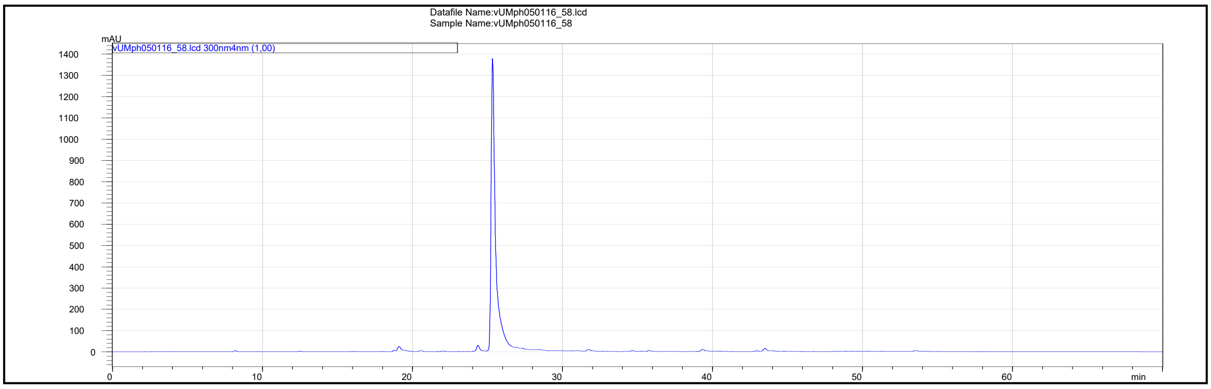

References:

1. Kukula-Koch, W.; Mroczek, T. Application of hydrostatic CCC-TLC-HPLC-ESI-TOF-MS for the bioguided fractionation of anticholinesterase alkaloids from Argemone mexicana L. roots. Anal Bioanal Chem 2005, 407, 2581-2589, DOI: 10.1007/s00216-015-8468-x.
2. Kukula-Koch, W.; Koch, W.; Angelis, A.; Halabalaki, M.; Aligiannis, N. Application of pH-zone refining hydrostatic countercurrent chromatography (hCCC) for the recovery of antioxidant phenolics and the isolation of alkaloids from Siberian barberry herb. Food Chem 2016, 203, 394-401, DOI: 10.1016/j.foodchem.2016.02.096.
3. Kukula-Koch, W.; Aligiannis, N.; Halabalaki, M.; Skaltsounis, A.L.; Glowniak, K.; Kalpoutzakis, E. Influence of extraction procedures on phenolic content and antioxidant activity of Cretan barberry herb. Food Chem 2013, 138, 1, 406-13. doi: 10.1016/j.foodchem.2012.10.045
